# Variable Intrafamilial Expression of ABCB4 Disease

**DOI:** 10.14309/crj.0000000000001113

**Published:** 2023-08-11

**Authors:** Lucia Zampaglione, Anne-Laure Rougemont, Laura Rubbia-Brandt, Marc Abramowicz, Michel Guipponi, Enrica Marchionni, McLin Valerie, Nicolas Goossens

**Affiliations:** 1 Division of Internal Medicine, Hôpital du Valais, Sion, Switzerland; 2 Division of Transplantation, Hôpitaux Universitaires de Genève, Geneva, Switzerland; 3 Division of Clinical Pathology, Hôpitaux Universitaires de Genève, Geneva, Switzerland; 4 Swiss Pediatric Liver Center, Pediatric Gastroenterology, Hepatology, and Nutrition Unit, Department of Pediatrics, Gynecology, and Obstetrics, University of Geneva, Geneva, Switzerland; 5 Division of Genetic Medicine, Hôpitaux Universitaires de Genève, Geneva, Switzerland; 6 Division of Gastroenterology & Hepatology, Hôpitaux Universitaires de Genève, Geneva, Switzerland

**Keywords:** ABCB4, cholestasis, phenotype, genotype, penetrance, transplantation

## Abstract

Progressive familial intrahepatic cholestasis type 3 (PFIC3) is a rare cholestatic liver disease with autosomal recessive inheritance caused by mutations in the *ABCB4* gene. The clinical presentation of PFIC3 varies significantly, displaying incomplete penetrance without clear genotype-phenotype correlations. As such, the suitability of living-related liver donation for children with advanced disease has been questioned. We report here the long-term follow-up of a patient with PFIC3 resulting in decompensated cirrhosis at 11 years who successfully underwent living donor liver transplantation from his father, who carried the same *ABCB4* homozygous mutation.

## CASE REPORT

**Figure 1.**
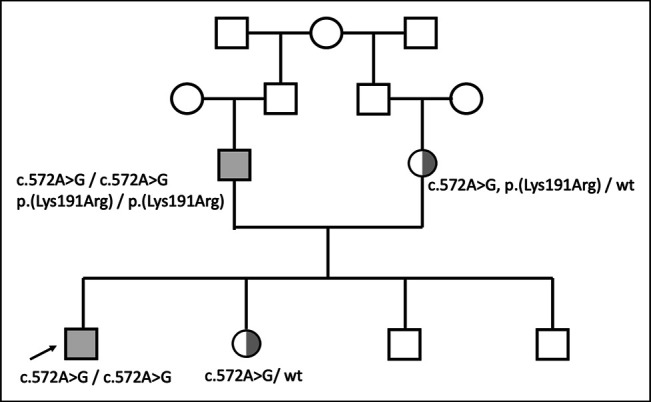
Pedigree displaying the known genetic distribution of the ABCB4 mutation within the family.

In 2008, a 10-year-old Libyan boy born to consanguineous parents (half first cousins) (Figure 1) was admitted after a minor motor vehicle accident. Liver function tests revealed moderate aminotransferase elevation (aspartate aminotransferase 178 U/L [N: <50]; alanine aminotransferase 11 U/L [N: <50]) and moderately elevated cholestasis markers (alkaline phosphatase 489 U/L [N: <102]; gamma-glutamyl transferase [GGT] 338 U/L [N: <40]; total bilirubin 35 μmol/L [N: <25] with conjugated bilirubin 15 μmol/L [N: <9.5]). Prothrombin time was 50%, international normalized ratio 1.39, factor V 64%, and low platelets (47 g/L [N: <350]), suggesting the presence of portal hypertension. Extensive workup was noncontributory. Imaging excluded biliary obstruction, and there was no history of hepatotoxic agents or herbal supplements.

Percutaneous liver biopsy showed chronic active hepatitis with cirrhosis; Mallory-Denk bodies and mild microvesicular steatosis were also seen. The histological characteristics, along with a significantly elevated fresh liver-fragment copper concentration (29 mmol/kg [N = 0.16–0.55]) and dry tissue concentration (1,845 μg/g [N = 10–35]), led to a tentative diagnosis of Wilson disease. Ophthalmological examination did not reveal Kayser-Fleischer rings and showed a nonspecific retinopathy. A transjugular intrahepatic portosystemic shunt was put in place, and treatment with D-penicillamine and ursodeoxycholic acid (UDCA) was initiated.

Despite an initial improvement in liver function, the presence of refractory encephalopathy at one-year follow-up led to the decision to evaluate the patient for liver transplantation. The patient did not qualify for deceased donation because he was not a resident; therefore, living donor options were explored. The patient's father, who was in good health and had a normal biological and imaging workup, underwent percutaneous liver biopsy for evaluation. Histology of the father's liver was unremarkable, revealing only minimal portal and lobular inflammation and mild predominantly macrovesicular steatosis affecting approximately 10% of hepatocytes, with no liver fibrosis. The father was thus selected as the living donor, and the patient was transplanted at 11 years with an uneventful postoperative course.

**Figure 2.**
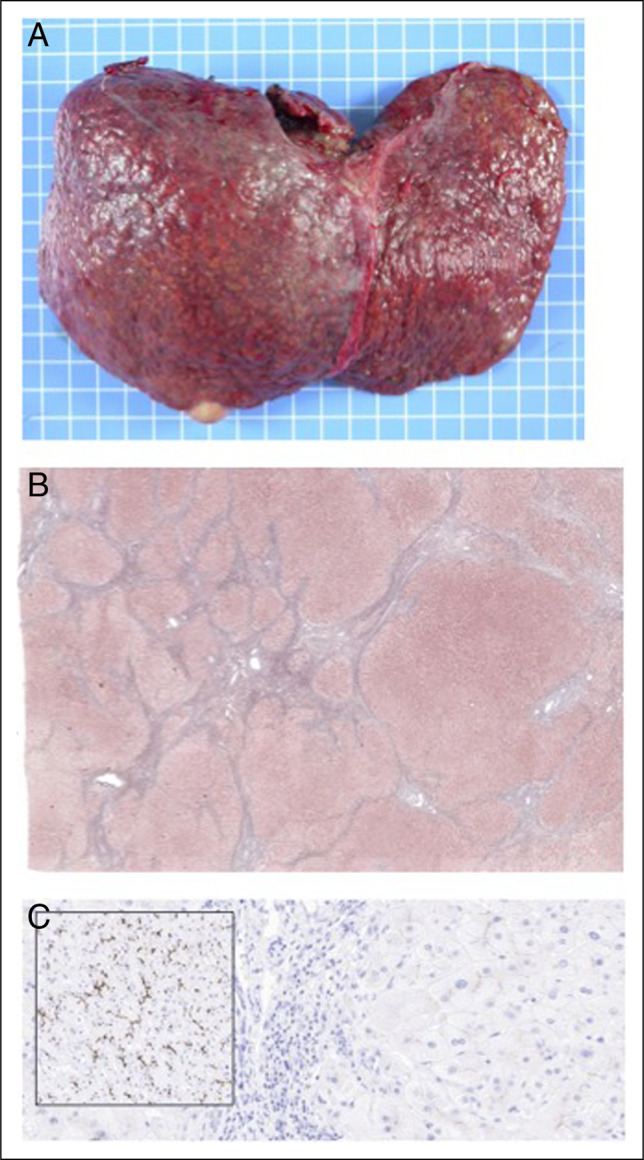
Liver explant at 12 years. (A, B) Liver cirrhosis (A, gross findings; B, histology, Masson trichrome stain). (C) Faint and focal canalicular reactivity to MDR3/ABCB4, contrasting with the strong BSEP/ABCB11 positivity (inset).

The liver explant (Figure 2) showed cirrhosis, and immunohistochemistry revealed only faint and focal reactivity to MDR3/ABCB4 (abcam mouse monoclonal, clone P3II-26, 1:150 dilution).

Persistent GGT elevation at 4 months after liver transplantation prompted liver biopsy, which revealed mild interlobular bile duct dystrophy. Subsequent workup excluded an early biliary anastomosis stenosis, and a wait-and-watch approach was adopted.

**Figure 3.**
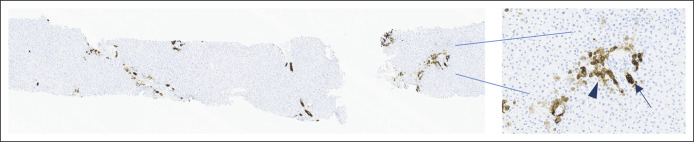
Liver biopsy, 8 years after liver transplant. CK7 showing rare dystrophic interlobular bile ducts (arrow) and focal aberrant reactivity in periportal hepatocytes (arrowhead), suggestive of chronic cholestasis.

Eleven years later, the 16-year-old sister presented with mild liver enzyme abnormalities followed by an acute hepatitis of unclear etiology. In addition, a detailed maternal history revealed severe pruritus during all pregnancies. Therefore, after obtaining informed consent, genetic testing was proposed to all family members. Whole-exome sequencing with a targeted gene panel analysis was performed on proband genomic DNA extracted from peripheral blood. The proband carried a homozygous c.572A>G; p.(Lys191Arg) *ABCB4* gene missense variant. Segregation analysis performed using Sanger sequencing showed that his father, the donor, carried the same homozygous variant. Both the sister and the mother were found to be heterozygotes (45 years and 17 years at diagnosis, respectively) consistently with their clinical signs. A definitive diagnosis of progressive familial intrahepatic cholestasis type 3 was made in the proband, and UDCA treatment was started. The most recent liver biopsy available at 8 years after transplantation revealed slowly evolving biliary dystrophy, with an absence of the interlobular bile duct in 3 of 7 available portal tracts (insufficient to meet the formal criteria of ductopenia, ie, loss of interlobular bile ducts in >50% of the portals, in a biopsy containing at least 10 portal tracts) and no significant inflammation or fibrosis. The bile duct alterations are highlighted by cytokeratin 7 (Dako mouse monoclonal, clone = V-TL 12/30, 1:100 dilution) that also shows focal aberrant hepatocyte reactivity consistent with chronic cholestasis (Figure 3).

To date, at 13 years after transplantation, the patient still presents with mild persistent elevation of cholestasis markers despite UDCA treatment (alkaline phosphatase 120 U/L [N: < 102]; GGT 119 U/L [N: < 40]).

## DISCUSSION

The more limited knowledge available on progressive familial intrahepatic cholestasis type 3 (PFIC3) and limited access to genetic testing at the time of transplantation led to a delayed diagnosis of the ABCB4 mutation in this case. In retrospect, the patient's clinical presentation, laboratory results, and histological data were all consistent with PFIC3 disease. The elevated copper concentration identified on the patient's liver biopsy 1 year before transplant is a common finding in patients with cholestasis and PFIC3 as a result of prolonged cholestasis.^1,2^ In the present day, we propose genetic testing for ABCB4 variants be performed if the indication for liver transplantation is high gGT cholestatic liver disease, if the donor biopsy shows signs of mild biliary disease, or in cases of relevant family history of cholestatic liver disease including intrahepatic cholestasis of pregnancy or low-phospholipid-associated cholelithiasis.

While the phenotypic diversity of *ABCB4* variants has been widely reported in the literature,^3–5^ this case further illustrates the intrafamilial phenotypic variability, variable expressivity, and wide-ranging clinical course of PFIC3 by demonstrating an uneventful long-term follow-up in a patient having received a graft carrying the same homozygous ABCB4 variant responsible for his end-stage liver disease. In this case, the graft followed the natural history of the donor liver, not of the recipient. The patient remains clinically stable at 13 years of follow-up, presenting only with mild bile duct alterations, which have evolved slowly over the course of several years. Based on this experience, we propose that ABCB4 mutations, even in the homozygous state, should not represent a formal contraindication to left lobe living liver donation, provided the preoperative liver biopsy of the donor is reassuring. However, an improved understanding of the natural history of patients with *ABCB4* variants is crucial to allow a better genotype-phenotype correlation, and an individualized approach to these patients, in particular in the context of potential living donor liver transplantation.

## DISCLOSURES

Author contributions: L. Zampaglione: writing of the manuscript and is the article guarantor. V. McLin and N. Goossens: cowriting of the manuscript and critical revision of the manuscript for important intellectual content. A. Rougemont, M. Abramowicz, M. Guipponi, and E. Marchionni: critical revision of the manuscript for important intellectual content.

Financial disclosure: None to report.

Previous presentation: Presented as a poster at SGG Annual Congress; September 15–16, 2022; Interlaken, Switzerland.

Informed consent was obtained for this case report.
